# Perspectives on Small Animal Radionuclide Imaging; Considerations and Advances in Atherosclerosis

**DOI:** 10.3389/fmed.2019.00039

**Published:** 2019-03-11

**Authors:** Eric J. Meester, B. J. Krenning, J. de Swart, M. Segbers, H. E. Barrett, M. R. Bernsen, K. Van der Heiden, Marion de Jong

**Affiliations:** ^1^Department of Radiology and Nuclear Medicine, Erasmus Medical Center, Rotterdam, Netherlands; ^2^Department of Biomedical Engineering, Thorax Center, Erasmus Medical Center, Rotterdam, Netherlands; ^3^Department of Cardiology, Thorax Center, Erasmus Medical Center, Rotterdam, Netherlands

**Keywords:** mice, nuclear imaging, SPECT, PET, atherosclerosis

## Abstract

This review addresses nuclear SPECT and PET imaging in small animals in relation to the atherosclerotic disease process, one of our research topics of interest. Imaging of atherosclerosis in small animal models is challenging, as it operates at the limits of current imaging possibilities regarding sensitivity, and spatial resolution. Several topics are discussed, including technical considerations that apply to image acquisition, reconstruction, and analysis. Moreover, molecules developed for or applied in these small animal nuclear imaging studies are listed, including target-directed molecules, useful for imaging organs or tissues that have elevated expression of the target compared to other tissues, and molecules that serve as substrates for metabolic processes. Differences between animal models and human pathophysiology that should be taken into account during translation from animal to patient as well as differences in tracer behavior in animal vs. man are also described. Finally, we give a future outlook on small animal radionuclide imaging in atherosclerosis, followed by recommendations. The challenges and solutions described might be applicable to other research fields of health and disease as well.

## Introduction

### Small Animal Radionuclide Imaging

Nuclear imaging using Single Photon Emission Computed Tomography (SPECT) or Positron Emission Tomography (PET) allows high-sensitivity and (semi-) quantitative imaging of physiological processes or molecular targets *in vivo*. Before clinical application, preclinical evaluation of novel radiotracers is a requisite to assess tracer characteristics such as *in vivo* tracer kinetics, target specificity, stability, and biodistribution. This is greatly facilitated by the wide-spread use of small animal models of disease as well as the development of state of the art small animal SPECT and PET systems, which allow tracer examination up to sub-mm resolution (1–6). However, preclinical nuclear imaging of small animals comes with a particular set of challenges and opportunities different from clinical nuclear imaging.

### Atherosclerosis

The challenges and opportunities of small animal imaging become apparent in e.g., atherosclerosis imaging. Atherosclerosis is an inflammatory disease in which fatty plaques might occlude an artery through continued lipid deposition or sudden rupture of vulnerable plaques. Occlusion of an artery can lead to myocardial infarction, stroke, or limb ischemia. Early detection and characterization of atherosclerosis is therefore important, but remains problematic. Many imaging techniques such as contrast enhanced Computed Tomography (CT) focus on degree of stenosis, but fail to identify vulnerable plaques. Functional imaging of biological processes involved in plaque development or progression may identify and localize plaques at risk of rupture. Moreover, the characteristics of a vulnerable plaque, such as the presence of intraplaque hemorrhage, a large influx of inflammatory cells, neovessel formation, or a thin fibrous cap (7), provides ample possibilities for nuclear imaging. Yet, when studying novel tracers that might fulfill this need, research teams are faced with challenges. Differences between animal models of atherosclerosis and the human pathophysiology can make imaging results difficult to interpret. Furthermore, the small size of the plaques in small animal models, as well as the low and diffuse density of targets in a plaque, can severely complicate the evaluation process including quantification options *in vivo*.

### Nuclear Imaging of Atherosclerosis

2-deoxy-2-[^18^F]fluoro-D-glucose ([^18^F]FDG) has been extensively studied for the detection and quantification of inflammatory cells in atherosclerosis (8, 9), and has been shown an independent predictor of recurrent events after stroke (10–12). Moreover, differentiation between different plaque phenotypes in the carotid arteries was successfully investigated using this tracer (13). However, unspecific uptake of [^18^F]FDG, especially in the metabolically active myocardium, limits its use to detect plaques in coronary arteries. As such more specific tracers are urgently needed.

In this review, we describe small animal radionuclide imaging with a strong focus on applications in atherosclerosis. We discuss differences between the pathophysiology of human and mouse atherosclerosis, related technical aspects, and challenges of small animal radionuclide imaging, as well as atherosclerosis tracer development and evaluation. Moreover, we discuss the future outlook and give recommendations.

## Considerations on Models of Atherosclerosis

### Animal Models of Atherosclerosis

A number of atherosclerotic animal models have been developed, as reviewed in Getz and Reardon (14). In short, porcine and primate models resemble human atherosclerosis best, yet are costly to maintain and are less established with regard to genetic modification. The plaques in rabbit models resemble human plaque less, as rabbit plaques mainly contain lipids. Rabbit models have certain advantages over mouse models, including the diameter of the abdominal aorta being similar to human coronary arteries and less subjected to movement. However, rabbit models are less frequently used since the introduction of the Apolipoprotein E deficient (ApoE^−/−^) and low density lipoprotein receptor knock-out (LDLR^−/−^) (KO) mouse models (15). Most atherosclerosis studies therefore use murine models, as mouse plaques develop faster than rabbit plaques, the mouse models are well-characterized, have low costs, and are widely available. Recent developments like clustered regularly interspaced short palindromic repeats/Cas9 (CRISPR/Cas9) targeted genome editing to create KOs (16), and pro-protein convertase subtilisin/kexin type 9 (PCSK9) injection to rapidly induce atherosclerosis (17), have created new opportunities in modeling human-like atherosclerotic disease in mice. We refer to Veseli et al. (18) for a more extensive review of mouse models of atherosclerosis. Besides advantages in using atherosclerotic mice, there are several considerations to be taken into account when choosing a mouse model and interpreting imaging results.

### Plaque Location and Composition

Like in humans, atherosclerosis in mice is multifocal and locates in specific regions of the vasculature, determined by the hemodynamic environment (19). Pre-clinical imaging studies generally study plaques located in the inner curve of the aorta, the carotid arteries, and brachiocephalic artery, while translating their results to human coronary disease. Plaque composition as well as plaque stability or vulnerability differ between mice and men; differences in lipid metabolism lead to different lipid profiles related to the ratio between high, very low, and low density lipoprotein (HDL, VLDL, and LDL) (14, 20). Moreover, thin caps or intraplaque hemorrhage are rare in traditional mouse models, whereas they are characteristic of human vulnerable atherosclerosis (21), and plaque rupture is rarely seen in commonly used mouse models (22). To create a mouse model with plaque rupture, double knock outs (23, 24) or invasive experimental interventions are required, which arguably do not mimic human plaque rupture mechanisms (25).

### Immune Subsets

Inflammatory cells are often used as imaging targets, because of the important role they have in plaque formation and progression. Yet, it is reported that human and mouse macrophage subsets differ (26, 27), which therefore makes validation in human tissue necessary.

Despite these differences between human and murine atherosclerosis, mice are valuable in testing radiotracers, as processes like angiogenesis and inflammation are present in mouse plaques. Therefore, mice can be used for proof of concept studies, or to assess tracer behavior *in vivo*. Moreover, *ex vivo* validation by gamma-counting, autoradiography, and immunohistochemistry allows better quantification of radiotracers. However, for reasons discussed above, translating results obtained in mouse models to expect human results should be done with caution.

## Technical Developments and Applications in Small Animal Radionuclide Imaging

### Nuclear Imaging of Mouse and Human Plaques

SPECT and PET can both provide very high sensitivity, even suitable for imaging of very small quantities of radiotracers (nM-pM range), enabling investigation of specific cells or pathophysiological processes. Developments in these systems for small animal imaging and in processing of imaging data allow better examination of novel radiotracers. Moreover, preclinical systems allow high resolution and sensitive examination of human tissues (28, 29). When imaging mouse atherosclerosis challenges become apparent: high spatial resolution is crucial in small murine plaques. The largest murine plaques are located in the aorta, which has a diameter of ~1 mm. High sensitivity is however also very important, as these small plaques contain relatively few target cells, on which receptor expression can be low compared to other disease models such as tumor models. Here we highlight a number of developments in imaging and image processing, see (30–36) for more extensive reviews on nuclear imaging methods.

### Preclinical SPECT

**Figure 1:**
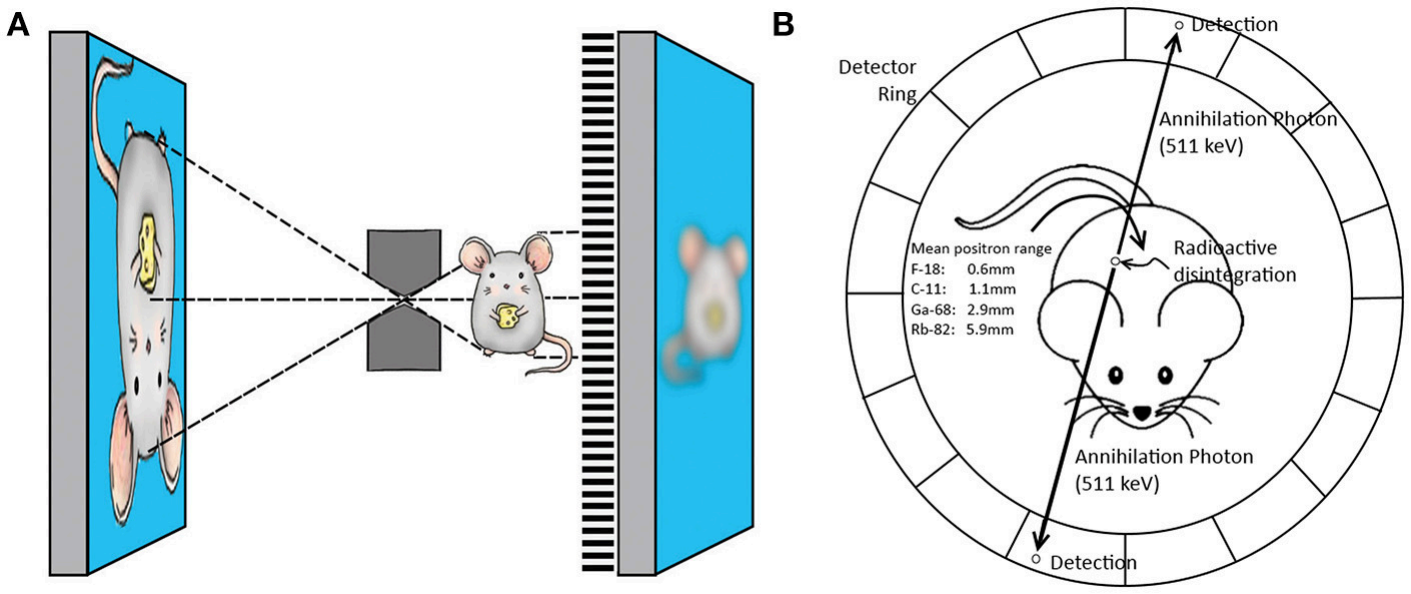
Panel **(A)** illustrates the principle of pinhole imaging. The collimator can be placed close to the source of radiation in preclinical imaging, resulting in a magnifying effect on the detector. The limited sensitivity is improved by using multiple pinholes and different pinhole geometries. Clinical SPECT mostly uses parallel hole collimators, which directly limits spatial resolution. Pinhole magnification can also achieve a higher spatial resolution for positron emitting isotopes In comparison to traditional coincidence PET (Image reproduced from thesis O. Ivashchenko, LUMC, ISBN 978-94-92516-35-0). Panel **(B)** shows the principle of PET coincidence detection. Two opposing detectors simultaneously measure a gamma photon providing the line along which the positron annihilated with an electron. This line does not coincide with the location the positron was emitted, because the positron travels a finite range before it annihilates. Especially for high energy positrons, e.g., ^68^Ga [mean positron range of 2.9 mm (46)], the positron range may limit spatial resolution in both pinhole PET and coincidence PET. Image adapted with permission from Fontaine et al. (47).

SPECT systems require a collimator to obtain directional information on gamma rays emitted from within the animal or patient sample to be imaged. Traditional clinical SPECT systems generally use a parallel hole collimator, which limits resolution and sensitivity in comparison to clinical PET systems that do not require a collimator (Table 1) (44). The choice of collimator heavily depends on the imaging task at hand because of the classic trade-off between resolution and sensitivity in collimator design. Regarding spatial resolution, major improvements have been made in preclinical SPECT by the introduction of pinhole cameras, in which magnified projection data can be acquired by choosing the right positions of the pinholes between the scintillation crystal and the animal (45), enabling sub-mm resolutions (Table 1 and Figure 1). Such high spatial resolutions can be achieved by decreasing the diameter of the pinhole, but come with the obvious trade-off of lower sensitivity. Multipinhole cameras combat the very low sensitivity of a single pinhole (39), and can reduce or even eliminate the need of rotating detectors or movement of the bed if only a small field of view (FOV) is required to answer the research question (48, 49). This greatly improves temporal resolution, offers the possibility of 3D gated imaging of the heart, and enables imaging of fast tracer kinetics (50). High sensitivity collimators have been developed (51), but the sensitivity of SPECT systems remains limited in comparison to that of PET because of the relatively low fraction of photons transmitted through the collimators.

**Table 1:** Shows a tabulated overview of properties of clinical and preclinical PET and SPECT.

	**Small Animal Scanners**		**Standard Clinical Scanners**	
	**Resolution [mm]**	Sensitivity ^**^ [%]	**Resolution [mm]**	**Sensitivity [%]**
SPECT ^99m^Tc	0.38–0.76 (37)	0.07–0.39 (37)	~10	~0.01
SPECT ^111^In	0.71-0.85 (37)	–	–	~0.01
Pinhole PET ^18^F	<0.85^*^ (38)	0.37 (38)	–	–
Coincidence PET ^18^F	1.61–2.34 (39)	1.19–6.72 (39)	6.4 (40)	1.33–2.29 (41)
Coincidence PET ^68^Ga	2.19 (42), 2.2 (43)	–	7 (40)	–

* Resolution was determined by visual assessment of a Jaszczak phantom instead of measuring the FWHM of a line source.

** Values for sensitivity should be interpreted with care, as no standard method exists to directly compare SPECT and coincidence PET sensitivity quantitatively. When covering a FOV the size of a PET FOV, the effective sensitivity of SPECT could well be several factors lower.

*Green colour indicates which modality performs better in a certain area, red indicates lower performance*.

### Preclinical PET

The sensitivity of PET scanners is at least an order of magnitude higher than SPECT cameras [>10 times (52), see Table 1], since no physical collimator is needed. In preclinical PET (ring diameter < 20 cm), the resolution is mostly limited by the positron range and the size of the detector elements. For low energy positron emitters (^18^F) both factors limit spatial resolution, for high energy positron emitters (^68^Ga) the positron range is the main limiting factor (40, 52, 53).

Positron emitting radionuclides can be imaged with a traditional coincidence based PET system and also with special pinhole collimation (54, 55). Traditional ring PET systems can achieve a better image quality in very low count rate studies, for higher count rate studies a multi-pinhole system may yield higher quality images due to the higher spatial resolution that can be achieved by pinhole magnification.

### Hybrid Imaging

Use of hybrid systems, providing an anatomical reference by (contrast-enhanced) CT or MRI (1, 2, 39, 56), are crucial in atherosclerotic mouse studies because the small plaques are located close to other tissues. MRI has the major advantage of providing soft tissue contrast, which is crucial to distinguish arteries from surrounding tissue. However, the better resolution and faster scanning time of CT make this method preferable in many instances, especially if contrast agents can be used. Moreover, CT provides direct means for attenuation correction (57), whereas an MR image is usually segmented into different tissue classes to obtain an estimate of the amount of attenuating material. New opportunities are opened by the combination of more modalities, such as optical tomography, or integrating PET and SPECT to allow dual-tracer imaging. Moreover, dual tracer imaging is also explored in PET (reviewed in Walrand et al. (58), allowing further possibilities in tracer imaging.

### Preclinical vs. Clinical Imaging

Preclinical SPECT can achieve a higher spatial resolution than preclinical PET platforms, whereas this is the other way round in clinical imaging (see Table 1). The higher resolution of preclinical SPECT often makes it the imaging method of choice for imaging of atherosclerotic mice because of the small sized plaques. Preclinical visualization of plaques with PET isotopes can further be complicated by positron range, as this can exceed the size of a plaque [e.g., ^68^Ga has a mean positron range of 2.9 mm (46)]. Image quality of clinical PET can be improved by Time of Flight (ToF), which reduces image noise by incorporating the time difference of the detected annihilation photon pair in the reconstruction. Clinical systems obtain a timing resolution of ~300 ps (59). In a preclinical system image quality did not improve for a timing resolution of 260 ps (60). Another difference comprises the small deviation from 180° between the annihilating photon pair (non-collinearity) that reduces the spatial resolution for systems with a larger PET ring diameter. This becomes a major limiting factor in clinical PET (52), whilst this effect is negligible in small animal PET. Also, in clinical practice gated imaging is used to improve image quality of moving structures like the heart and its coronary arteries (61, 62). A trade-off has to be made between scan time and image quality to obtain sufficient count statistics in each gate. Using image registration techniques, motion-free static images can be obtained without affecting count statistics (63). This application is thus far not commonly applied in preclinical imaging. Finally, the high sensitivity and simultaneous acquisition of all projection angles in whole body PET makes it superior over SPECT with regard to temporal resolution, as the time needed to obtain sufficient counts directly determines scanning time.

### Image Reconstruction

Virtually all preclinical and clinical images are reconstructed by an iterative reconstruction algorithm. These algorithms rely on a model of the physics in the imaging process, where improvement of the model improves the quality of the reconstructed images. For example, spatial resolution can be improved by including the point spread function in the model (64). Monte Carlo based methods can improve scatter estimation and can include depth of interaction effects for PET in the iterative reconstruction (65, 66). Efficient algorithms can reduce reconstruction time while preserving image quality even in low count studies (67).

### Quantification

Besides visualizing the radiotracer distribution, most atherosclerosis imaging studies perform (semi-) quantitative Volume Of Interest (VOI) or voxel based measurements. This is expressed in percentage injected dose per gram, standardized uptake value, or target to background ratio (%ID/g, SUV, or TBR). It is important to consider against which background a target tissue is visualized. Plaque to blood ratio is usually a useful TBR in atherosclerosis imaging, as blood signal can interfere with plaque signal. The myocardium would be a suitable background when using a radiotracer such as [^18^F]FDG in the coronary arteries. Images can be quantified when applying a suitable predetermined calibration factor to convert counts per volume to activity per volume (Bq/ml). Attenuation and scatter correction is less important in preclinical imaging due to the smaller amount of attenuating material, but their application still improves quantification accuracy (57, 68–70). When imaging structures with sizes around or below the resolution of the camera, like plaques in mice, it is important to realize that partial volume effects can cause a substantial underestimation of the true value (71, 72). This makes absolute quantification accuracy dependent on the imaging task. Numerous compensation techniques for partial volume effects have been described (73), but none have been validated or used in preclinical arthrosclerosis imaging yet.

## Radiotracers and Their Targets

### Radiotracers and Radionuclides

Radiotracers should target processes relevant to disease, which in atherosclerosis are e.g., inflammation, endothelial dysfunction, neovascularization, hypoxia, cell death, or microcalcification. Moreover, the target should ideally be abundantly expressed and specifically localized in plaques and not in surrounding tissues. Also, blocking studies should be performed, as non-specific uptake in the arterial wall could complicate plaque visualization. Radiotracers need to be stable *in vivo* without pharmacological or toxic effects, and should be labeled with an appropriate radionuclide, matching the pharmacokinetics of the tracer. Radiotracers labeled with short-lived PET radionuclides should have a fast clearance to prevent blood signal from interfering with plaque visualization. Moreover, it is advantageous if radiotracers show rapid diffusion into tissues. If a radiopharmaceutical is being developed with the objective of use in humans, then the radionuclide intended for human use should be used in the animal studies if at all possible as this will simplify translation of preclinical data. In some cases, however, the use of a different radionuclide for some of the preclinical studies is unavoidable or even preferable, as it can be preferred to label radiotracers with SPECT radionuclides for high-resolution preclinical evaluation vs. PET radionuclides for clinical use.

### Beyond [^18^F]FDG

**Figure 2:**
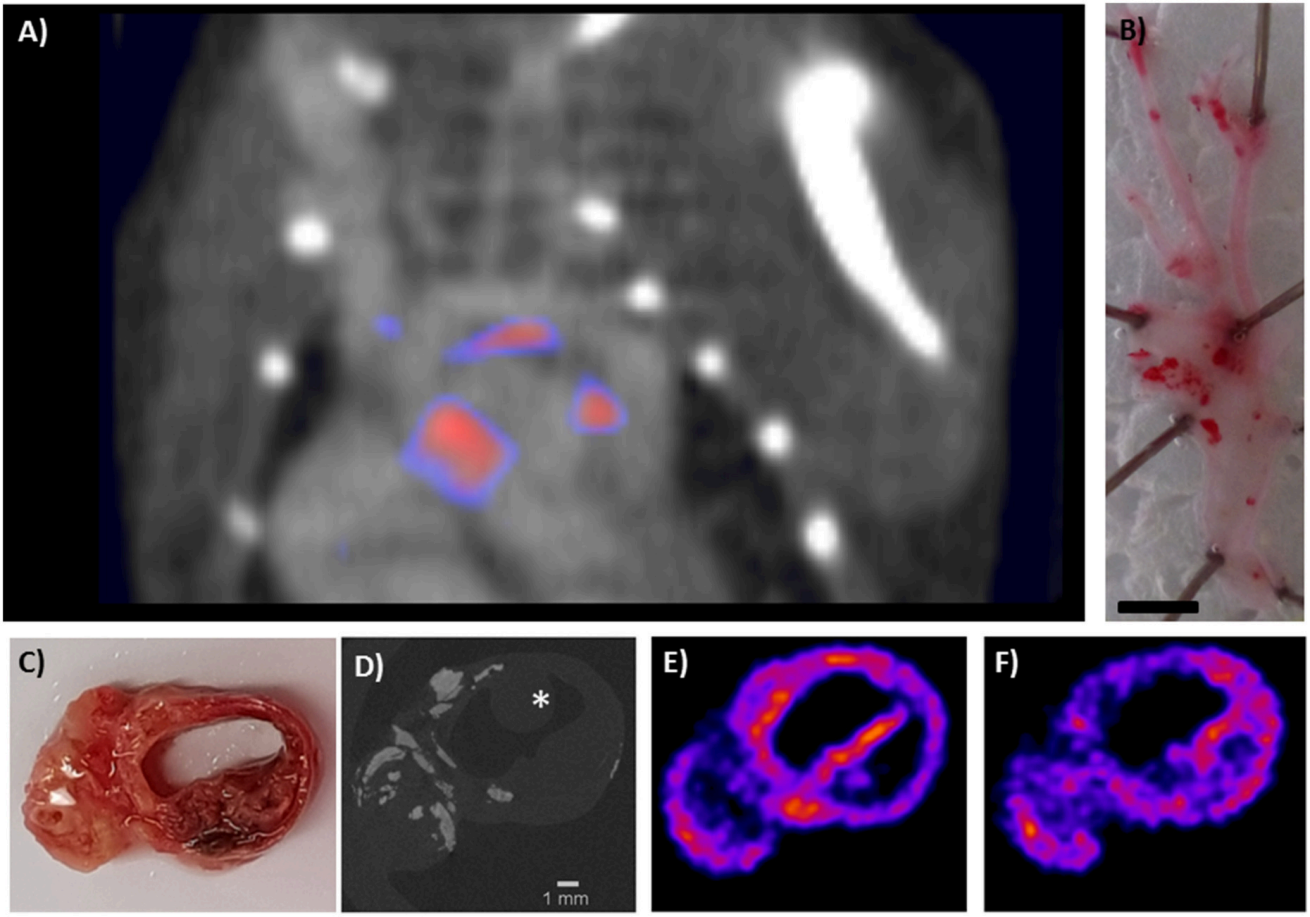
Two cases which exemplify the opportunities and challenges in preclinical imaging using multi-pinhole collimators. Panel **(A)** shows a contrast enhanced SPECT/CT scan of the thoracic region of an ApoE^−/−^ mouse (on 20 weeks high fat diet), imaged with [^111^In]In-DANBIRT, which targets leukocytes via Leukocyte Function associated Antigen 1 (LFA-1). LFA-1 is expressed in a high-affinity state on leukocytes near regions of inflammation, and can therefore be used to visualize inflamed plaque. The image shows uptake in plaque regions in the inner curve of the aortic arch and near the aortic leaflets. These common sites of plaque formation in this mouse model are visible in the excised, opened Oil Red O stained artery of an ApoE^−/−^ mouse on the right **(B)**. Panel **(A)** shows the high resolution which can be achieved with preclinical SPECT, considering the mouse aorta is ~1 mm in diameter. This case also illustrates some of the challenges in preclinical imaging as the small size of the plaque and the presence of few target cells require a state of the art imaging system with high resolution and sensitivity. Moreover, the recommended injection dose of 20 μL contrast agent per 5 g bodyweight (Exitron nano 12000) can be challenging, as the combined injection volume of contrast agent and radiotracer injection can easily exceed the recommended injection volume for mice, which can have adverse effects on the animal health and experimental outcome. Reduction of the injection volume of the radiopharmaceuticals can be achieved by using smaller tubing during radiolabelling. The timing of injection is also important, as blood signal of radiotracers can be high after injection, yet the amount of activity reduces with radionuclide half-life. Moreover, many contrast agents circulate a limited period in the vasculature. Optimization before an experiment, considering the dose and timing of injection, is therefore crucial. In this example, we injected 50 MBq (200 pmol) [^111^In]In-DANBIRT 2 h before SPECT imaging, and the contrast agent directly at the start of CT imaging. Scale bar = 2 mm [reproduced from Meester et al. (87), no permissions required]. **(C–F)** depict an example of a high resolution dual-isotope preclinical SPECT/CT scanning protocol applied to diseased human arterial tissue. Examination of the local differences in dual-radiotracer uptake with respect to the atherosclerotic pathophysiology was performed on **(C)** a human carotid endarterectomy slice of 2 mm thickness, which was incubated for 60 min with [^111^In]In-DANBIRT (targeting leukocytes) and [^99m^Tc]Tc-DEMOTATE (targeting activated macrophages; both 1 nmol, 100 MBq/nmol). [^99m^Tc]Tc-DEMOTATE targets somatostatin receptor subtype 2, which is expressed on activated macrophages. **(D)** Functional plaque morphology was resolved with high resolution μCT (15 min scan, full scan angle, 0.24 mA, 50 kV, 75 ms, 500 μm reconstructed resolution), where calcifications are denoted by the bright white regions. The asterisk (*) marks the sample holder. μCT was co-registered to SPECT (90 min scan) reconstructions of **(E)** [^111^In]In-DANBIRT and **(F)** [^99m^Tc]Tc-DEMOTATE. The two radioisotopes can be separated by selecting the correct energy windows for the photon peaks of ^111^In and ^99m^Tc (^111^In photopeaks 171 and 245 keV, energy windows 158–187 keV and 219–267 keV. ^99m^Tc photopeak 140 keV, energy window 125–152 keV). This hybrid functional imaging approach can be used to gain greater insights into radiotracer uptake in diseased tissues. Plaque status can be assessed via the presence of calcifications, whereas [^111^In]In-DANBIRT and [^99m^Tc]Tc-DEMOTATE ascertain inflammatory status by visualizing total inflammation and activated macrophages, respectively. Such scans could lead to a better risk stratification of atherosclerotic patients. It is interesting to see the different distribution patterns of these inflammation-targeted tracers within the plaque, which indicates that plaque detection alone gives only limited information when making a risk stratification of atherosclerotic patients. The timing of imaging is important as the radionuclides have different half-lives, and correct separation of the photon peaks requires sufficient counts to be acquired. Another challenge is to examine which incubation time allows the radiotracers to diffuse into the tissue, while keeping tissue degradation at a minimum (Courtesy H.E.B, Erasmus MC).

[^18^F]FDG PET has shown major promise in atherosclerosis imaging (8). [^18^F]FDG, being a glucose analog, is taken up by metabolically active cells such as macrophages in plaque, and can therefore be used for PET imaging of atherosclerosis. Plaque inflammation can be quantified using [^18^F]FDG, plaques can be monitored over time, and the effect of treatment can be visualized (74). However, unspecific myocardial uptake of [^18^F]FDG limits the applicability of imaging in coronary artery disease. Therefore, novel radiotracers targeting different disease processes with a higher specificity are being developed and evaluated. Table 2 lists a number of radiotracers and their targets tested in preclinical *in vivo imaging* studies in the past 10 years, and potential clinical follow up studies. Figure 2 includes 2 cases in which the possibilities and challenges of small radionuclide imaging of atherosclerosis are exemplified. Reference (125) reviews older studies performed with PET.

**Table 2:** Shows radiotracers applied in a selection of preclinical *in vivo* atherosclerosis imaging studies from 2008 to 2018, and mentions potential clinical follow-up studies.

**Disease characteristic**	**Target**	**Ligand**	**Radionuclide**	**Animal studies**	**Clinical studies**
Inflammation	Macrophages	FDG	^18^F	(13)	(11) retrospective, *n* = 513
	Macrophages, SST2	DOTATATE	^68^Ga	(75)	(76) retrospective, *n* = 70 (77) Prospective, *n* = 20 (78) Prospective, *n* = 42
	Macrophages, MR	FDM Tilmanocept	^18^F ^111^In	(79) (80)	
	Macrophages, FR	EC20 ECO800 FOL	^99m^Tc ^111^In ^18^F	(81) (82) (83)	
	Macrophages, CXCR4	Pentixafor	^68^Ga	(84)	(85) Retrospective, *n* = 38 (86) Retrospective, *n* = 51
	Leukocytes, LFA-1	DANBIRT	^111^In	(87)	
	Macrophage proliferation	FLT	^18^F	(88)	
	Chemokine receptors	DOTA-vMIP-II	^64^Cu	(89, 90)	
		DOTA-DAPTA	^64^Cu	(91)	
	LOX-1	Liposome-LOX-1	^111^In	(92)	
		Camelid antibody fragment	^99m^Tc	(93)	
	TSPO	PK11195 Ge-180	^11^C ^18^F		(94) Prospective, *n* = 15 (95) Prospective, *n* = 32 (96)
	Macrophage phagocytosis	TNP Macroflor	^64^Cu ^18^F	(97) (98)	
Apoptosis	Apoptosis and Necrosis	AnxAF568 Hypericin	^99m^Tc, ^124^I	(99)	
	Apoptosis	Duramycin	^99m^Tc	(100)	
	Apoptosis	Duramycin and Annexin V	^99m^Tc	(101)	
Angiogenesis	α_v_β_3_ integrin	NC100692	^99m^Tc	(102)	
		NOTA-RGD	^68^Ga	(103)	(103) Prospective, *n* = 4
		Flotegatide	^18^F	(104)	
		Galacto-RGD	^18^F	(105)	(106) Prospective, *n* = 10
		NOTA-3-4A	^64^Cu	(107)	
		Maraciclatide	^99m^Tc	(108)	
		IDA-D-[c(RGDfK)]_2_	^99m^Tc	(109)	
	VEGF 1 and 2	scV/Tc	^99m^Tc	(110, 111)	
Proteolysis	MMP activation	RP805	^99m^Tc	(112, 113)	
		RP782	^111^In	(114, 115)	
	GPVI	GPVI-fragment crystallized	^64^Cu	(116)	
Endothelial activation	P-selectin	P-selectin antibody	^64^Cu	(117)	
		Fucoidan	^68^Ga	(118)	
	VCAM-1	cAbVCAM1-5	^99m^Tc ^18^F	(119–121) (122)	
		4V	^18^F	(123)	
Hypoxia	Redox	FMISO	^18^F	(124)	

*SST2, somatostatin receptor subtype 2; MR, Mannose Receptor; FR, Folate Receptor; CXCR4, C-X-C Chemokine Receptor type 4; LFA-1, Leukocyte Function associated Antigen-1; LOX-1, oxidized LDL receptor 1; TSPO, Translocatio Protein; VEGF, Vascular Endothelial Growth Factor; MMP, Matrix Metalloprotease; GPVI, Platelet Glycoprotein VI; VCAM-1, Vascular Cell Adhesion Molecule-1*.

Currently, [^68^Ga]Ga-Pentixafor (84, 85), [^68^Ga]Ga-DOTATATE (75, 78), and [^18^Na]NaF (reviewed in Mckenney-drake et al. 9) show very promising results in patients. Recent successful mouse studies have been performed on other tracers such as [^111^In]In-DANBIRT (87), [^111^In]In-Tilmanocept (80), or [^99m^Tc]Tc-Maraciclatide (108). Direct comparisons between radiotracers as performed in Rinne et al. (75), are lacking however, which makes it difficult to see where radiotracers can complement each other, or which radiotracer is most suitable for different aspects of plaque visualization.

## Perspectives and Recommendations

### Risk Stratification in Atherosclerosis

The development of non-invasive imaging techniques visualizing atherosclerosis and particularly vulnerable plaque is a major aim in cardiovascular imaging (126). The individual and societal impact of such imaging tools can be substantial. They could contribute to current risk stratification, which is based on conventional cardiovascular risk factors and non-traditional risk factors such as biomarkers and coronary artery calcium score. Recent clinical trials focus on the importance of anti-inflammatory strategies for treatment of cardiovascular disease (127, 128). Biomarkers (e.g., hsCRP, IL-6) are mostly used for assessment of inflammation, and might be complemented by non-invasive molecular imaging of arterial inflammation in guiding treatment with these new anti-inflammatory drugs. Novel tracers therefore could provide extra prognostic value, and aid in further risk-stratification by identifying plaques at risk and patients in need of treatment.

### Crossing Borders

Diagnostic imaging tools developed for other (non-cardiac) diseases such as oncology have been shown to be of significance in atherosclerosis research (129). Somatostatin receptor imaging using ^68^Ga-DOTATATE, developed for diagnosis of neuro-endocrine tumors, has been validated as a novel marker of atherosclerotic inflammation via overexpression of the somatostatin receptor subtype 2 (SST2) on activated macrophages. This has led to better discriminating power of high risk coronary lesions compared to [^18^F]FDG (75, 78). Similarly, imaging of macrophages with ^68^Ga-Pentixafor also originates from oncology (84, 85). Furthermore, technical challenges in image post-processing in atherosclerosis might be improved by developments from other research fields (130, 131). Vice versa, research on other diseases can benefit from our increased knowledge, as diagnosis of other inflammatory diseases such as arthritis can be difficult and hampered by similar challenges encountered in atherosclerosis.

## Conclusion

Developments in animal models and imaging systems have facilitated and enhanced the opportunities for small radionuclide imaging and will likely continue to do so in the foreseeable future. These advances have been essential in preclinical imaging of atherosclerosis, which requires high resolution and sensitivity, and has resulted in a large number of novel radiotracers being evaluated. This allows ample opportunity for clinical translation, where more insight into atherosclerosis, as well as relevant imaging targets, are highly required.

## Data Availability

The datasets generated for this study are available on request to the corresponding author.

## Author Contributions

All authors listed have made a substantial, direct and intellectual contribution to the work, and approved it for publication.

### Conflict of Interest Statement

The authors declare that the research was conducted in the absence of any commercial or financial relationships that could be construed as a potential conflict of interest.
